# Editorial: Applications of digital twin technology in dentistry

**DOI:** 10.3389/fbioe.2025.1624734

**Published:** 2025-06-11

**Authors:** Sandipan Roy, Raphaël Richert, João Manuel R. S. Tavares, Pierre Lahoud

**Affiliations:** ^1^ Department of Mechanical Engineering, SRM Institute of Science and Technology, Kattankulathur, India; ^2^ Faculté d’Odontologie Université Lyon 1, Lyon, France; ^3^ INSA Lyon, CNRS, LaMCoS, UMR5259, Villeurbanne, France; ^4^ Hospices Civils de Lyon, PAM Odontologie, Lyon, France; ^5^ Faculty of Engineering, University of Porto, Porto, Portugal; ^6^ Division of Periodontology and Oral Microbiology, Department of Oral Health Sciences, Faculty of Medicine, KU Leuven, Leuven, Belgium; ^7^ OMFS-IMPATH Research Group, Department of Imaging and Pathology, University Hospitals Leuven, Leuven, Belgium; ^8^ Department of Conservative Dentistry and Periodontology, LMU University Hospital, LMU Munich, Munich, Germany

**Keywords:** digital dentistry, digital twin, in-silico, dentistry, biomechanics

The merging of computational mechanics, biomedical engineering and digital health has catalysed a paradigm move in current dentistry. At the forefront of this transformation is the application of Digital Twin Technology (DTT) – a virtual representation that reflects the structure, function, and responses of physical dental systems. Built on simulation-driven design, DTT uses real-time patient data, imaging, and finite element modeling to optimize diagnosis, treatment planning, and biomechanical evaluation. The content of this research topic explores the integration of digital twin concepts into contemporary orthodontic and prosthodontic interventions, highlighting how such simulations are advancing accuracy, reducing invasiveness, and informing next-generation clinical workflows.

Among the most influential applications of digital twin technology is its use in clear aligner therapy (CAT), where finite element models are used to replicate the biomechanical behavior of teeth under different aligner protocols. Zhang et al. used a recursive finite element method to simulate long-term orthodontic tooth movement and evaluate the intrusion pattern of anterior teeth during retraction using clear aligners in the extraction area (Zhang et al.). Their digital twin revealed how specific infiltration techniques can reduce the “roller-coaster” effect–a complex, non-linear movement of the teeth during space closure. The study introduces a novel overtreatment planning approach based on strong biomechanical evidence rather than clinical heuristics.

Based on this premise, Li et al. explored how aligner thickness and tooth movement techniques affect the outcome of maxillary arch expansion **(** Li et al. **)**. Their three-dimensional simulations highlight that substitute posterior tooth movement can lead to higher growth efficiency, although this must be stable with periodontal health risks. The research displays how digital twins can simulate different movement sequences and aligner parameters to monitor modified tissue-friendly expansion plans.

Beyond orthodontics, digital twins have a significant impact on implant dentistry. Alshadidi et al. developed *in silico* models to examine the effects of surface texturing and hybrid coatings on the biomechanical behavior of dental implants under conditions of variable bone quality **(** Alshadidi et al. **)**. The digital twin framework offers mechanistic understandings into implant design and personalized implant selection. This study highlights how virtual models assess load-bearing response and simulate interactions at the implant-bone interface, capturing remodeling processes essential for long-term success.

In the pitch of oral surgery, Xing et al. applied a digital twin of the tooth-periodontal ligament (PDL) complex to inspect the biomechanical rationale behind torsion-based extraction of single-rooted teeth **(** Xing et al. **)**. Usually, rotational extraction was discouraged for flat or curved-root teeth due to apparent fracture risk. Nevertheless, through finite element modeling, this study exposed how anatomical variables such as root curvature, width, bone type, and root length influence the optimal torsion angle (OTA) compulsory for safe extraction. Clinically validated, their digital twin model provides a biomechanical basis for expanding the suggestions for torsion-based extractions–a breakthrough in the field of minimally invasive dentistry.

Degradation of orthodontic attachments is an essential factor in aligner retention and force application in finite element studies **(** Li et al. **)**. The digital twin simulated different wear conditions across two attachment types–rectangular and original control–during canine distalization. Results showed that surface wear significantly reduced movement efficiency, especially at the primary control attachment, indicating the need for timely clinical monitoring and rebonding. This study demonstrates how digital twins can predict treatment deviations arising from component wear, increasing the predictability of aligner-based treatment.

Digital twin technology has progressively been used in dentistry to simulate anatomical, material, and procedural variables precisely. This is bridging the gap between preclinical and clinical insights. However, challenges remain, such as assuming isotropic material properties and static boundary conditions.

For clinical acceptance, the inclusion of patient-specific biological responses and the standardization of modeling protocols are crucial. The creation of FE models as digital twins marks a significant advancement, ushering in an era of hyper-personalization characterized by predictive and adaptive treatment plans.

The content of this research topic emphasizes the transformative impact of digital twin technology in dental science and clinical practice. Integrating high-fidelity simulation with biological insights not only promises improved outcomes but also provides new standards in personalized, minimally invasive, and efficient dental care. As digital dentistry evolves, the research presented here provides both a blueprint and a beacon for future innovations.

